# Persistent fever, neck swelling, and small vessel vasculitis following tonsillectomy in a patient with Behçet’s disease: a case report

**DOI:** 10.1186/1752-1947-6-371

**Published:** 2012-10-30

**Authors:** Claudia Wagner, Dominik Schär, Marianne Tinguely, Isabelle Kunz

**Affiliations:** 1 University Hospital of Zurich, Clinic of Internal Medicine, Zürich, Switzerland; 2 Clinic of Immunology, Zürich, Switzerland; 3 Institute of Surgical Pathology, Rämistrasse 100, Zürich, 8091, Switzerland

## Abstract

**Introduction:**

Behçet’s disease commonly presents with recurrent oral and genital mucocutaneous ulcerations, uveitis and various skin manifestations. Other clinical symptoms include gastrointestinal ulcerations, arthritis, venous thrombosis, arterial aneurysms and central nervous system affection. Vasculitis underlies most clinical symptoms of Behçet’s disease.

**Case presentation:**

We report the case of a 62-year-old European Caucasian woman with Behçet’s disease who presented with persistent fever and neck soft-tissue swelling, despite broad antibiotic treatment, two weeks after acute tonsillitis and a tonsillectomy. Diffuse epi- and mesopharyngeal swelling shown on a computed tomography scan of her neck and persistently elevated serum markers of inflammation initially prompted suspicion of an infectious etiology. Magnet resonance imaging of her neck and a neck tissue biopsy finally confirmed small vessel vasculitis involving skin, subcutaneous tissue and muscle. Considering the clinical presentation, past medical history and histological findings, we interpreted our patient’s symptoms as a flare of Behçet’s disease. Immunosuppressive treatment led to rapid clinical improvement.

**Conclusion:**

A patient with Behçet’s disease developed small vessel vasculitis of the soft tissue of her neck after tonsillitis and a tonsillectomy. Infection and surgery probably triggered a flare of Behçet’s disease.

## Introduction

Behçet’s disease (BD) is a relapsing chronic inflammatory multisystem disease with a wide spectrum of clinical features. BD is highly prevalent in young adults from Mediterranean, Middle Eastern and East Asian countries; its geographical distribution follows the Silk Road. Clinical features of BD mostly occur as mucocutaneous oral and genital ulceration, uveitis, arthritis and various cutaneous manifestations: ulcers of the skin, papulopustulosa, erythema nodosum, subcutaneous nodules and cellulitis. BD is also associated with gastrointestinal, neurologic and vascular disease [1]. Large vessel disease and central nervous system involvement mostly occurs in young males and is associated with higher disease-related mortality rates [2].

There are no pathognomonic laboratory tests for BD and the diagnosis is based solely on clinical symptoms. Diagnostic criteria of BD according to the International Criteria for BD (ICBD 2006) are listed in Table 1. The pathergy phenomenon, a hypersensitivity reaction of skin tissues to trauma, is pathognomonic for BD. A positive pathergy test is defined as the occurrence of a small red bump or pustule, one to two days after applying a needle prick on the forearm [1].

**Table 1:** Diagnostic criteria of Behçet’s disease

**Major criteria**	
Recurrent oral ulcerations	Aphthous ulceration, ≥ three episodes within 12 months
**Minor criteria**	
Recurrent genital ulceration	Aphthous ulceration
Eye lesions	Anterior or posterior uveitis/retinal vasculitis
Skin lesions	Erythema nodosum-like, papulopustular, pseudofolliculitis
Positive pathergy test	Small red bump or pustule, 24 to 48 hours after applying a needle prick on the forearm

Most clinical manifestations of BD are believed to be from vasculitis because histological examination of affected tissue in patients with BD most frequently reveals vascular inflammation [3]. BD may involve blood vessels of all sizes on both the arterial and venous sides of the circulation. Small vessel vasculitis is found in cutaneous, gastrointestinal, retinal and central nervous system lesions. Large vessel vasculitis usually presents as relapsing venous thrombosis or thrombophlebitis and less commonly as aneurysms of medium and large arteries [4].

The etiology of BD is still unknown, although various genetic and environmental factors have been related to disease susceptibility. Human leukocyte antigen B51 has been shown to play a role in the pathogenesis of BD [3]. Infectious agents such as streptococci and herpes simplex virus have been suggested as infectious triggers of BD in genetically susceptible individuals. Streptococcal antigens are believed to induce auto-inflammatory reactions in patients with BD via cross-reactivity with human proteins [5]. Systemic exposure to streptococcal antigens during streptococcal skin testing in clinical studies [5] and during dental procedures [6] has been shown to trigger BD flares.

## Case presentation

A 62-year-old European Caucasian woman presented at our emergency department with fever, sore throat and painful swallowing for four days. Clinical findings at presentation included inarticulate speech, left-sided tonsil and soft-palate swelling and enlarged left cervical lymph nodes. Her past medical history revealed that BD had been diagnosed eight years earlier. At that time, our patient presented with oral and genital ulcerations, painful metacarpal swelling of her ring finger, thrombophlebitis and painful lower leg swelling and erythema. Treatment at that time had consisted of high-dose methylprednisolone, cyclophosphamide and colchicine. The course of the disease had been oligosymptomatic with a single episode of relapse with oral and genital ulcerations, seven years earlier. Maintenance treatment with colchicine and azathioprine was discontinued one year earlier. Co-morbidities were insulin-dependent diabetes mellitus and osteoarthritis of her left knee following anterior cruciate ligament surgery.

Our patient was immediately referred to otolaryngology for treatment of an assumed peritonsillar abscess. A pharyngeal incision yielded no pus. Antibiotic treatment with clindamycin was started. Computed tomography (CT) of her neck showed inflammation of the left peritonsillar tissue, but no peritonsillar abscess. A bilateral tonsillectomy was performed the next day. Histological examination showed acute phlegmonous inflammation of her left tonsil. There was no streptococcal (or any bacterial) growth in throat cultures or in the removed tonsillar tissue. During the following weeks, our patient had persistent fever up to 39°C and painful swelling of her neck. Compression of her upper airways caused moderate dyspnea and a daily dose of 125mg methylprednisolone was administered from day three onwards for one week, to reduce the swelling. Her C-reactive protein level was persistently elevated (range 200 to 400mg/L). On day seven, a repeat CT of her neck showed diffuse pharyngeal swelling but no abscess. Imipenem was added as a broad-spectrum antibiotic to prevent parapharyngeal and mediastinal spreading of a presumed infection. Our patient did not respond to treatment and her fever and neck swelling persisted.

**Figure 1:**
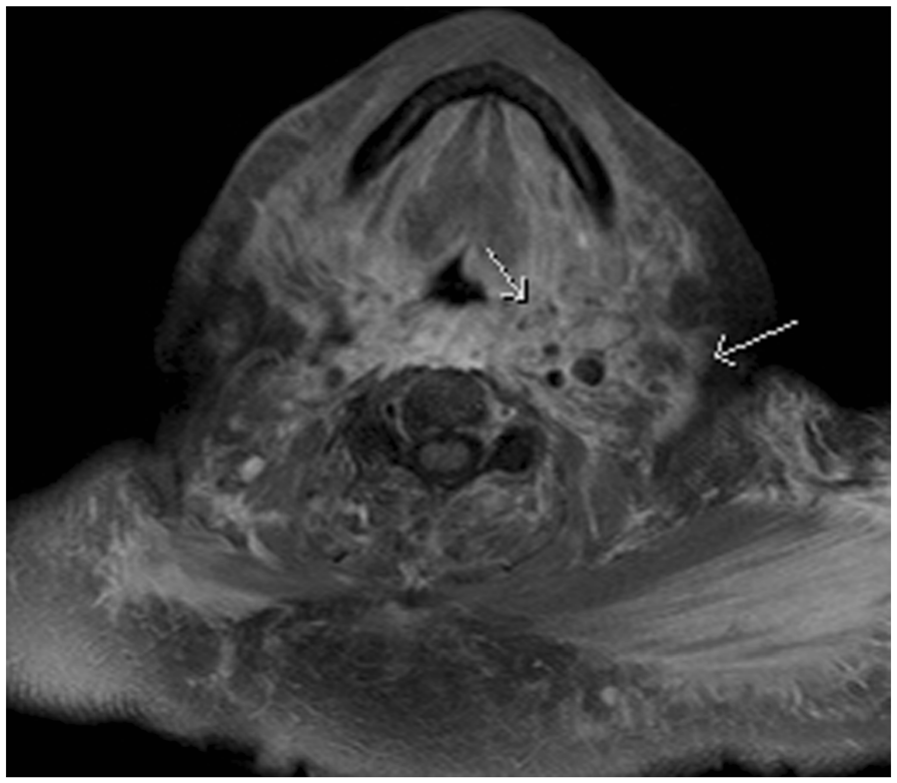
**Magnetic resonance imaging of the neck.** Magnetic resonance imaging of the neck on day 20 showed diffuse, predominantly left-sided inflammatory infiltration of epi- and mesopharyngeal tissues (arrows).

**Figure 2:**
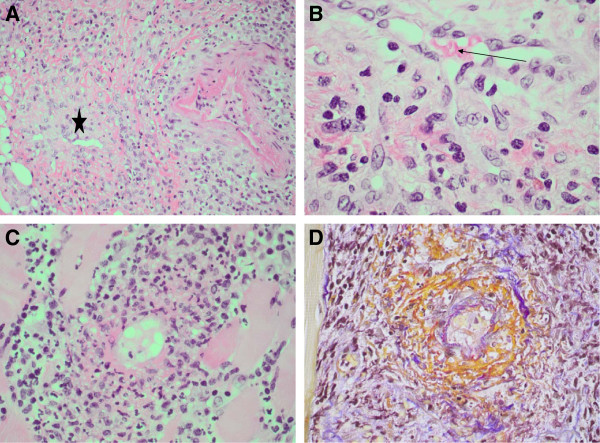
**Spectrum of inflammation activity in small vessels of the subcutaneous biopsy.** (**A**) Cross-section of two vessels with different stages of inflammation; one with endothelial thickening (asterisk) and the second with a mixed perivascular inflammatory infiltrate consisting of lymphocytes, plasma cells, eosinophilic granulocytes, accompanied by apoptotic figures. (**B**) Small vessel wall with transmural inflammation as described in (**A**) (arrow: vessel lumen with erythrocytes). (**C**) Leukocytoclastic vasculitis with karyorrhexis and fibrinoid necrosis in a small vessel wall. (**D**) Same vessel as in (**C**) showing the fibrinoid necrosis (orange) in an acid fuchsin orange G stain.

On day 15, our patient was referred to our internal medicine ward. A CT scan of her neck, chest and abdomen ruled out superior vena cava syndrome, pulmonary embolism and thoracic or abdominal abscess. On day 17, she newly developed single, non-palpable petechial palmar and plantar lesions on her right hand and foot. Transthoracic echocardiography showed no signs of endocarditis. Blood and urine cultures tested negative. Syphilis and human immunodeficiency virus were ruled out. A vasculitis screening (antineutrophil antibodies, antineutrophil cytoplasmic antibody, urinary sediment) was negative. On day 20, magnetic resonance imaging of her head and neck showed diffuse, predominantly left-sided inflammatory infiltration of epi- and mesopharyngeal tissues (Figure 1). A transcutaneous biopsy involving skin, subcutis and sternocleidomastoid muscle tissue confirmed leukocytoclastic small vessel vasculitis and perivasculitis, compatible with a relapse of BD (Figure 2).

Our patient showed rapid clinical and laboratory improvement upon treatment with methylprednisolone 1g daily for three days. The prednisone dose was then reduced to 1mg/kg body weight over three weeks and azathioprine was concomitantly up-titrated to a daily dose of 200mg over four weeks. Because of the side effects of azathioprine, the treatment was switched to mycophenolate mofetile (daily dose of 1g) after three months. Prednisone was tapered to 5mg over the course of one year. To date, our patient has experienced no recurrence of BD under the immunosuppressive maintenance therapy.

## Discussion

We report this case because the presentation of BD in our patient was uncommon. Our research of the literature yielded no similar case of small vessel vasculitis of neck soft tissue, although there have been reports of head and neck swelling caused by superior vena cava syndrome in patients with BD [7].

Our patient had diffuse neck swelling but no local skin manifestation that could have prompted vasculitis in the first place, except for single non-palpable petechial palmar and plantar lesions that developed only later on during the disease course. Although our patient had a history of BD, she had been in disease remission without treatment for one year. In addition, her disease had previously presented with more classic symptoms, such as oral and genital ulcers and skin lesions. Persistently elevated inflammation markers suggested systemic infection. CT and magnetic resonance imaging of her neck showed diffuse inflammation of all tissues. Because our patient initially presented with tonsillitis, there was concern about an infection of the parapharyngeal space. Broad-spectrum antibiotics were administered for three weeks to prevent spreading of the infection and carotid involvement in this high-risk patient with diabetes. The results of a tissue biopsy, which showed leukocytoclastic and fibrinoid vasculitis and perivasculitis of small vessels and capillaries, along with her past medical history and clinical presentation were highly suggestive of a relapse of BD. The main differential diagnosis was medication-induced hypersensitivity vasculitis. However, our patient did not have typical palpable purpura, noticeably absent on her neck, and a macular or urticarial rash was also absent.

We believe that both tonsillitis and tonsillectomy triggered small vessel vasculitis in the surrounding neck soft tissue in our patient. Oral and pharyngeal infections and procedures have been shown to play an important role in triggering BD in patients with a genetic predisposition [5,6]. A positive correlation has been demonstrated between the treatment of caries and periodontitis and the short-term occurrence of oral ulcers in patients with BD. However, fewer oral ulcers occurred during long-term follow-up of patients with BD who underwent dental procedures [6].

Surgical procedures have been shown to trigger symptoms in perioperative tissue of patients with BD by a pathergy-like effect. Pathergy-like phenomena have been described in patients with BD after surgery of the gastrointestinal tract (as gastrointestinal ulcers at the operative site) and after vascular surgery (presenting as localized vasculitis and aneurysm) [4]. There are only a few case reports of BD disease flares triggered by head and neck surgery. A case of neuro-Behçet disease was reported after a tooth extraction [8]. Another patient with BD developed myositis of the calf after a tonsillectomy [9].

Perioperative immunosuppression has been shown to improve outcome after vascular and gastrointestinal surgery in patients with BD [10,11]. However, there are currently no treatment guidelines on immunosuppressive treatment for patients with BD in disease remission or with minor disease manifestations before elective surgery [12].

## Conclusion

Tonsillitis and a tonsillectomy triggered small vessel vasculitis of the neck soft tissue in our patient. The diagnosis was delayed due to an uncommon disease presentation and was eventually confirmed by tissue biopsy. BD is known for its variable clinical manifestations and disease flares can be triggered by oropharyngeal surgery. Whether preoperative immunosuppression would have prevented disease activation in our patient remains unclear.

## Consent

Written informed consent was obtained from the patient for publication of this case report and the accompanying figures. A copy of the written consent is available for review by the Editor-in-Chief of this journal.

## Competing interests

The authors declare that they have no competing interests.

## Authors’ contributions

CW and IK were the treating physicians on the internal medicine ward where our patient was hospitalized. They wrote the manuscript and were responsible for the revision of the manuscript. DS was the consultant immunologist. He was involved in the diagnosis and treatment of Behçet’s disease. MT was responsible for the histological diagnosis in this patient and provided the histological images presented. All authors read and approved the final manuscript.
